# Genetic dissection of rice grain shape using a recombinant inbred line population derived from two contrasting parents and fine mapping a pleiotropic quantitative trait locus *qGL7*

**DOI:** 10.1186/1471-2156-11-16

**Published:** 2010-02-26

**Authors:** Xufeng Bai, Lijun Luo, Wenhao Yan, Mallikarjuna Rao Kovi, Wei Zhan, Yongzhong Xing

**Affiliations:** 1 National key laboratory of crop genetic improvement and National Center of Plant gene Research (Wuhan), Huazhong Agricultural University, Wuhan 430070, China; 2 Shanghai Agrobiological Gene Center, 2901 Beidi Road, Shanghai 201106, China; 3 College of Forestry, Northwest A&F University, Yangling, 712100, China

## Abstract

**Background:**

The three-dimensional shape of grain, measured as grain length, width, and thickness (GL, GW, and GT), is one of the most important components of grain appearance in rice. Determining the genetic basis of variations in grain shape could facilitate efficient improvements in grain appearance. In this study, an F_7:8 _recombinant inbred line population (RIL) derived from a cross between *indica *and *japonica *cultivars (Nanyangzhan and Chuan7) contrasting in grain size was used for quantitative trait locus (QTL) mapping. A genetic linkage map was constructed with 164 simple sequence repeat (SSR) markers. The major aim of this study was to detect a QTL for grain shape and to fine map a minor QTL, *qGL7*.

**Results:**

Four QTLs for GL were detected on chromosomes 3 and 7, and 10 QTLs for GW and 9 QTLs for GT were identified on chromosomes 2, 3, 5, 7, 9 and 10, respectively. A total of 28 QTLs were identified, of which several are reported for the first time; four major QTLs and six minor QTLs for grain shape were also commonly detected in both years. The minor QTL, *qGL7*, exhibited pleiotropic effects on GL, GW, GT, 1000-grain weight (TGW), and spikelets per panicle (SPP) and was further validated in a near isogenic F_2 _population (NIL-F_2_). Finally, *qGL7 *was narrowed down to an interval between InDel marker RID711 and SSR marker RM6389, covering a 258-kb region in the Nipponbare genome, and cosegregated with InDel markers RID710 and RID76.

**Conclusion:**

Materials with very different phenotypes were used to develop mapping populations to detect QTLs because of their complex genetic background. Progeny tests proved that the minor QTL, *qGL7*, could display a single mendelian characteristic. Therefore, we suggested that minor QTLs for traits with high heritability could be isolated using a map-based cloning strategy in a large NIL-F_2 _population. In addition, combinations of different QTLs produced diverse grain shapes, which provide the ability to breed more varieties of rice to satisfy consumer preferences.

## Background

Rice is one of the most important cereal crops and staple foods in Asia. According to its grain shape, rice is primarily classified into long, medium, and short categories, by which the ratios of grain length (GL) to grain width (GW) are more than 3.0, between 2.1 and 2.9, and smaller than 2.0, respectively http://www.ams.usda.gov/AMSv1.0/getfile?dDocName=STELDEV3003761. People from North and South America, Southern China, and Europe usually prefer rice with long and slender grains, whereas those from Japan, Northern China, and North and South Korea prefer rice with short and round grains [1,2]. Juliano and Villareal reviewed grain quality of world rice types and reported that the correlations between GW and cooked rice hardness were significant in 18 countries/locations [3]. In addition, Yang et al. and Luo et al. also mentioned that cooking quality is associated with grain shape [4,5]. However, difference in cooking quality among the three shape categories is mainly determined by the chemical components and texture of their grains. In most cases, long grain rice has a high grain amylose content and after cooking, it is often firm and fluffy (not sticky); medium grain rice has a low amylose content and after cooking, it is often soft, moist, and sticky in texture. The cooking quality and amylose content in short grain rice are similar to those of rice in the medium grain category.

Grain size is usually evaluated by 1000-grain weight (TGW), which is one of the three key components, along with grain number per panicle (GPP) and panicle number per plant, contributing to rice grain yield and is positively correlated with grain shape traits including GL, GW, and grain thickness (GT) [6,7]. Grain shape has attracted significant attention in rice breeding programs due to its contributions to rice yield and quality [8].

Grain shape has been widely accepted as a complex trait controlled by multiple genes with small effects. Extensive efforts to determine the genetic basis of grain shape have used forward and reverse genetic strategies. Initially, studies focused on characterizing mutants and the expression of major genes associated with grain size, for example, the *Lk-f *gene, which confers long kernel size [9], or *Mi*, which confers short kernel size [10]. However, finding mutants of most grain shape genes in nature is not easy. Alternatively, quantitative trait locus (QTL) analysis based on genome wide mapping is a good strategy that has been widely used for mapping rice grain shape genes during the last 20 years. Previous studies used different populations, such as F_2_, F_3_, backcross, double-haploids, and recombinant inbred lines (RILs) http://www.gramene.org/qtl. Among these, the RIL population has particular advantages including repeatability, which favors the genetic analysis of quantitative traits because experiments can be replicated over years and locations. Combined with several fine genetic maps developed in recent years, many QTLs for grain shape have been identified. Tan et al., using F_2:3 _and RIL populations, detected two major QTLs for GL and GW on chromosomes 3 and 5, respectively. Several minor QTLs were also detected [11]. The two major QTLs for grain shape were also commonly detected in the same intervals on chromosomes 3 and 5, respectively, across different populations [7,11-15].

To date, thousands of QTLs have been detected in primary rice mapping populations. For fine mapping and cloning these QTLs, it is vital to make a population in which the targeted QTLs behave as the characters of a single mendelian factor. Therefore, an advanced population such as a near isogenic line population (NIL), which minimizes the noise of genetic background, is a prerequisite for QTL map-based cloning. Recently, increasing numbers of NILs have been used for QTL fine mapping and cloning. In terms of grain shape, Li et al. reported fine mapping of *gw3.1 *in the pericentromeric region of chromosome 3 using NILs, which is derived from a tropical *japonica *cultivar as the recurrent parent backcrossed to a wild rice [16]. Wan et al. found QTL *qGL-3 *for GL and *qGW-5 *for GW using chromosomal segment substitution lines (CSSLs) derived from the cross Asominori × IR24 [14]. Interestingly, these two QTLs were repeatedly identified in eight different environments. Fan et al. finely mapped the *GS3 *locus, a major QTL for GL and GW, and determined its candidate gene using advanced populations [17]. Based on these related reports, Zhang suggested that GL is mostly controlled by the *GS3 *locus on chromosome 3 and GW is largely conditioned by *GS5 *on chromosome 5 [18]. So far, one GL QTL, *GS3 *(encoding a transmembrane protein), two GW QTLs, *GW2 *(encoding RING-type E3 ubiquitin ligase) and *qSW5*/*GW5 *(whose biochemical function remains unclear), have been cloned [17,19-21]. In addition, Xie et al. finely mapped two TGW QTLs, *gw8.1 *and *gw9.1*, and concluded that there were significant correlations between TGW and grain shape [22,23].

Consecutive backcrossing is the conventional strategy for developing NILs and has been extensively used in mapping QTLs. Although molecular marker-assisted selection (MAS) can aim to any genome region, advanced backcross QTL-NIL development is both laborious and time consuming. An efficient method for a rapid NIL development is to seek the heterogeneous inbred family (HIF) at a QTL region [24]. The basic work flow for HIF-based NIL (HIF-NIL) development is first to screen the RIL heterozygous in the target QTL (high generation, eg, F_5 _or F_6_), second to evaluate the variation of target trait phenotype, and finally, to get the NILs (seeds harvested from the heterozygotes at the targeted genome region). Therefore, HIF-NIL development is much more efficient and more rapid for either major or minor QTLs as compared to the conventional strategies.

In this study, an RIL population derived from a cross between two rice cultivars, Nanyangzhan and Chuan7, was used for QTL mapping for grain size and grain shape. An NIL-F_2 _population of minor QTLs was constructed. The objectives of this study were (1) to detect QTLs for GL, GW, and GT, (2) to evaluate the power of QTL detection for grain shape as compared with other reports, and (3) to fine map a minor QTL, *qGL7*, with pleiotropic effects on GL, TGW, and spikelets per panicle (SPP).

## Methods

### Mapping population and field experiment

A total of 185 RILs were derived from the cross between *japonica *rice Nanyangzhan and *indica *rice Chuan7 by single-seed descent. Nanyangzhan has large grains, whereas Chuan7 has small grains. The RILs F_7 _and F_8 _were planted in a bird-net-equipped field on the experimental farm of Huazhong Agricultural University in the 2006 and 2007 rice-growing seasons in Wuhan, China. Field trials were carried out following the randomized complete block design with two replications within each year. At about 25 days old, 10 seedlings of each line were transplanted into one row in the main field with a distance of 16.5 cm between the plants within a row and 26.4 cm between rows. Eight plants in the middle of each row were harvested individually to score the traits.

Based on QTL mapping results from 2006, 185 RILs were screened for heterozygotes at the markers flanking the QTLs. In RIL76, the interval between RM22065 and RM5720 exhibited heterozygosity, but was homozygous in other genetic backgrounds. Selfing of RIL76 produced progeny segregating for *qGL7 *population in a near isogenic background. The *qGL7 *NIL-F_2 _population of 201 individuals was planted in Wuhan during the summer and their F_3 _families of 16 plants each were grown in Hainan during the winter of 2007.

### Trait measurement

Twenty full-filled rice grains were chosen randomly from each plant for trait measurement. GL was estimated by placing 20 grains one by one in a straight line along a ruler. These 20 seeds were individually measured for GW and GT using an electronic digital caliper (Guanglu Measuring Instrument Co. Ltd, China) with a precision of 0.1 mm. The averaged GL, GW, and GT of 20 grains as the trait values of that line were used for data analysis. The grain length-to-width ratio (LWR) is equal to GL divided by its GW. SPP was measured as the total number of spikelets per plant divided by its panicle number. TGW was calculated as the grain weight per plant divided by its grain number multiplied by 1000.

### DNA makers and linkage map construction

Fresh leaves were collected from 185 RILs (F_7_) individually for DNA extraction. DNA was extracted using a microisolation method as described by Cho et al. with minor modifications [25]. Polymorphic simple sequence repeat (SSR) markers between the parents Nanyangzhan and Chuan7 were used to genotype the population. All polymorphic markers were used for mapping the RIL population. The SSR assay was carried out essentially as described by Wu and Tanksley [26]. The genetic linkage map with RILs was constructed by using MAPMAKER/EXP version 3.0b [27]. The local linkage map based on the NIL-F_2 _population was developed. Genetic distance was calculated in Haldane function [28]. According to the sequence alignment between *japonica *cultivar Nipponbare and *indica *cultivar 93-11, three primer pairs for three InDel markers were designed with software "*Primer3*" for fine mapping *qGL7 *(Table 1).

**Table 1:** Basic information for three self-developed InDel markers

Markers	Physical location	Prod. size*	Anneal temperature	Primers	
				
				Forward (5'-3')	Reverse (5'-3')
RID76	28.303 Mb	149	58°C	caccgaagactgatcagcaa	tcacattcgagtggagcaac
RID710	28.420 Mb	170	58°C	catgttatcgtgtgggcttt	tctgttgctgcagctgaact
RID711	28.482 Mb	138	58°C	gcacatgcatgctaggacat	agccggtaaatttcttgcac

*Production size in bp was predicted from the sequence of *japonica *variety Nipponbare.

### Data analysis

Heritability for the traits GL, GW, and GT was calculated based on the experiments using the formula: , where ,  and  were the estimates of genetic, G by E and error variances derived from the mean square expectations of two-way ANOVA, n = 2 being the number of environments, and r = 2 being the number of replicates.

QTL analysis in RILs was performed by composite interval mapping (CIM) using the computer program Windows QTL cartographer 2.5 [29]. Window size was set as 10 cM and stepwise regression analysis was used to detect cofactors. The QTL main effects were estimated using the maximum-likelihood estimation method. The logarithm of odds (LOD) threshold at the experiment-wise significance level of 0.05 was determined by computing 1000 permutations of each morphologic character. The LOD threshold ranged from 2.8 to 3.1. In the *qGL7 *NIL-F_2 _population, MAPMAKER/QTL 1.1b software was used for QTL analysis. Walking speed for QTL scanning was 1 cM per step.

Two-way ANOVA was conducted to analyze digenic interaction between QTLs in the computer program Statistica 7.0 [30].

## Results

### Grain shape variation in the RIL population

**Figure 1:**
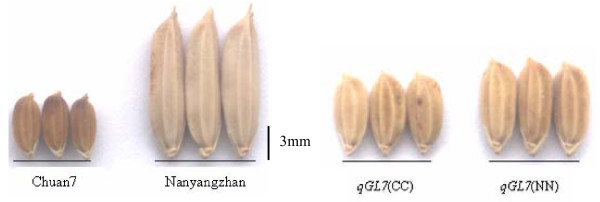
**Rice grains of both parents and two *qGL7 *homozygous NILs**. *qGL7 *(NN) and *qGL7 *(CC) mean the NIL lines with Nanyangzhan and Chuan7 homozygous alleles at *qGL7*, respectively.

**Figure 2:**
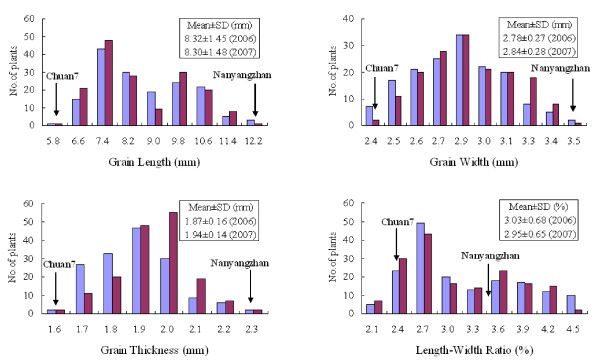
**Frequency distributions of grain shape traits in the RIL population**. Blue bars and dark red bars indicate 2006 and 2007, respectively.

A highly significant difference for all four investigated traits was observed between the two parents, Nanyangzhan and Chuan7 (Figure 1). Nanyangzhan has a typical large grain size of 43.3 g/1000 grains with 12.2 mm in GL, 3.5 mm in GW, and 2.3 mm in GT. Chuan7 is a variety with an extremely small grain size of 11.4 g/1000 grains; it possesses low trait values of 5.8 mm in GL, 2.4 mm in GW, and 1.6 mm in GT. GL variation was wide, ranging from 5.8 to 12.2 mm; frequency distribution showed a bimodal pattern with GL 9.0 mm as the boundary for each year (Figure 2). GW and GT exhibited normal distribution patterns in the 2 years. No transgressive segregation was observed for any of the traits except LWR (Figure 2).

### Heritability and correlation

GL, GW, and LWR showed high broad heritability of 97%, 91%, and 95%. GT was comparatively low, but its heritability still reached 78%. GL was positively correlated with GT and LWR in both years. GW was negatively correlated with GL and LWR, but positively correlated with GT (Table 2).

**Table 2:** Correlation coefficients among the four traits

Trait	GL	GW	GT	LWR
GL		-0.23**	0.27**	0.89**
GW	-0.17*		0.56**	-0.63**
GT	0.42**	0.59**		-0.03
LWR	0.89**	-0.59**	0.08	

The correlation coefficients in 2006 and 2007 are below and above the diagonal, respectively

*, **Significant at the level of *α *= 0.05 and 0.01, respectively

### Genetic linkage map

**Figure 3:**
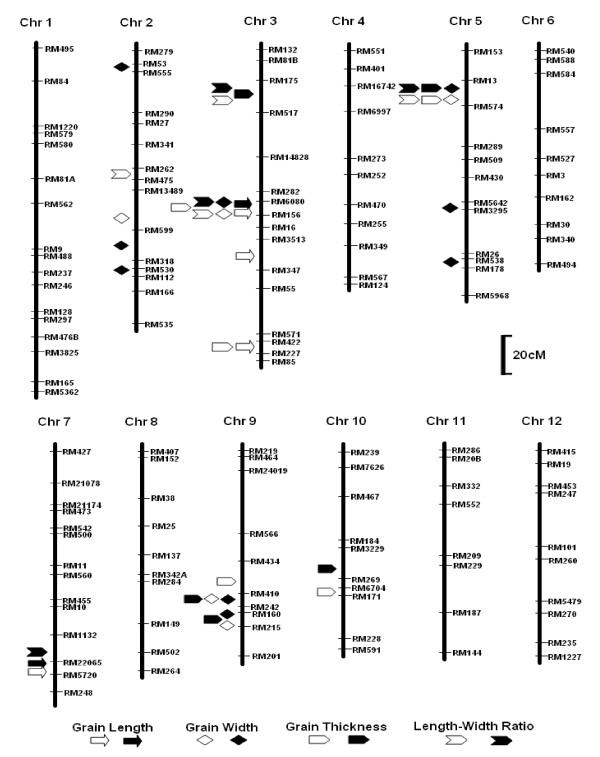
**Genetic linkage map showing QTL positions detected in the RIL population**. Black and white shapes indicate 2006 and 2007, respectively.

A total of 502 SSR markers were used for screening polymorphic markers between the parents Nanyangzhan and Chuan7. Of these, 262 showed polymorphism between parents. Finally, 164 evenly distributed polymorphic markers were selected for genotyping the RIL population. A genetic linkage map covering a 1635.9-cM genome region was constructed (Figure 3). The average distance between adjacent markers was 9.9 cM.

### QTL for grain shape traits

#### GL

Four QTLs for GL were detected on chromosomes 3 and 7 in both years (Table 3). *qGL3a *and *qGL7 *were commonly detected in both years. *qGL3b *and *qGL3c *were detected only in 2007. Nanyangzhan alleles appeared at three QTLs but *qGL3b *increased GL. The major QTL, *qGL3a*, in the marker interval between RM6080 and RM156 having a LOD score of more than 46, explained more than 65% of the phenotypic variance in both years. The other QTL individually explained a small GL variation of less than 5%. These three QTLs were located on chromosome 3 and were loosely linked with a distance of more than 20 cM from each other (Figure 3).

**Table 3:** QTLs for grain shape detected in the RIL population derived from the cross between Nanyangzhan and Chuan7

			2006			2007		
				
Traits	QTL	Interval	LOD	A^a^	V^b ^%	LOD	A^a^	V^b ^%
GL	*qGL3a*	RM6080-RM156	46.2	1.20	65.8	53.4	1.30	72.2
(mm)	*qGL3b*	RM3513-RM347				4.4	-0.30	4.1
	*qGL3c*	RM422-RM227	2.3^c^	0.22	2.4	5.7	0.30	4.9
	*qGL7*	RM22065-RM5720	4.7	0.30	5.1	5.4	0.30	4.7
GW	*qGW2a*	RM53-RM555	3.8	0.05	4.1			
(mm)	*qGW2b*	RM13489-RM599				6.0	0.10	15.6
	*qGW2c*	RM599-RM318	4.7	0.08	9.3			
	*qGW2d*	RM530-RM112	4.3	0.06	5.9			
	*qGW3*	RM6080-RM156	6.2	-0.08	8.9	3.4	-0.07	7.0
	*qGW5a*	RM13-RM574	21.0	0.17	38.1	14.0	0.17	36.3
	*qGW5b*	RM5642-RM3295	5.8	0.09	11.3			
	*qGW5c*	RM538-RM178	5.8	0.08	7.8	2.3^c^	0.06	5.3
	*qGW9a*	RM410-RM242	5.9	0.07	7.5	3.6	0.07	5.6
	*qGW9b*	RM160-RM215	6.6	0.08	9.2	5.1	0.08	8.4
GT	*qGT3a*	RM175-RM517	3.0	0.06	12.5	2.1^c^	0.04	7.4
(mm)	*qGT3b*	RM6080-RM156				3.3	0.04	7.3
	*qGT3c*	RM422-RM227				3.1	0.03	6.1
	*qGT5*	RM13-RM574	3.7	0.05	9.1	8.9	0.06	19.1
	*qGT9a*	RM434-RM410				3.1	0.03	5.3
	*qGT9b*	RM410-RM242	4.3	0.05	9.4			
	*qGT9c*	RM160-RM215	8.9	0.07	17.1	2.6^c^	0.03	5.2
	*qGT10a*	RM3229-RM6704	3.4	0.04	6.1			
	*qGT10b*	RM6704-RM171				4.4	0.04	10.4
LWR	*qLWR2*	RM262-RM475				3.8	-0.14	3.7
	*qLWR3a*	RM175-RM517	4.2	0.19	6.0	3.4	0.22	8.9
	*qLWR3b*	RM6080-RM156	48.0	0.50	57.0	51.3	0.53	64.2
	*qLWR5*	RM13-RM574	13.4	-0.25	13.5	5.5	-0.16	5.3
	*qLWR7*	RM1132-RM22065	3.2	0.13	3.5			

^a^A, additive effect, positive additive effect means Nanyangzhan allele increasing trait values.

^b^V, variance explained by the QTL.

^c^QTL with LOD ≥ 2.8 in one year but 2.0 ≤ LOD ≤ 2.8 in the other year are also listed.

#### GW

Ten QTLs were detected for GW (Table 3). One, two, three, and four QTLs were detected on chromosomes 3, 9, 5, and 2, respectively (Figure 3). Only four QTLs (*qGW3*, *qGW5a*, *qGW9a *and *qGW9b*) could be commonly detected in both years. A major QTL, *qGW5a*, was located in the marker interval between RM13 and RM574 with a high LOD score of more than 14 and explained more than one third of the GW variance in both years. *qGW9a *and *qGW9b *were located in the linked region about 15 cM away. *qGW5a*, *qGW5b*, and *qGW5c *were sparsely located on chromosome 5. *qGW2a *was far away from *qGW2b*, *qGW2c*, and *qGW2d*, which were located in a neighboring interval. Of the 10 QTLs, Nanyangzhan alleles at the other nine QTLs except for *qGW3a *had a positive effect on GW.

#### GT

Nine QTLs were identified for GT (Table 3 and Figure 3). Nanyangzhan alleles of all the nine QTLs increased GT. Only the major QTL, *qGT5*, was commonly detected in both years and explained 9.1% and 19.1% of phenotypic variation, respectively. The other eight QTLs were detected only in one year. Three linked QTLs and three sparsely distributed QTLs were identified on chromosomes 9 and 3, respectively. *qGT10a *and *qGT10b *were mapped to the neighboring regions in 2006 and 2007, respectively. Although *qGT9c *with a large LOD score of 8.9 could explain 17.1% of phenotypic variation in 2006, it was not detected in 2007.

#### LWR

Five QTLs were located on four chromosomes (Table 3 and Figure 3). Two QTLs were detected on chromosome 3 in both years. Nanyangzhan alleles increased LWR. *qLWR5 *was observed in both years, but Nanyangzhan alleles decreased LWR. *qLWR2 *and *qLWR7 *were detected only in one year. The major QTLs, *qLWR3b *and *qLWR5*, shared the same intervals with major QTLs, *qGL3a *and *qGW5a*, respectively.

In summary, 28 QTLs were found for the four grain shape traits. Three QTL hotspot regions were found on chromosomes 3, 5, and 9. No QTL for grain shape was identified on chromosomes 1, 4, 6, 8, 11, and 12.

### Digenic interaction

QTL genotypes were replaced with close-linked marker genotypes when QTL-by-QTL digenic interaction was analyzed. All possible digenic interactions among all 28 QTLs were analyzed by two-way ANOVA. However, no significant digenic interaction was observed at the level of P = 0.01%.

### Validation of *qGL7*

**Figure 4:**
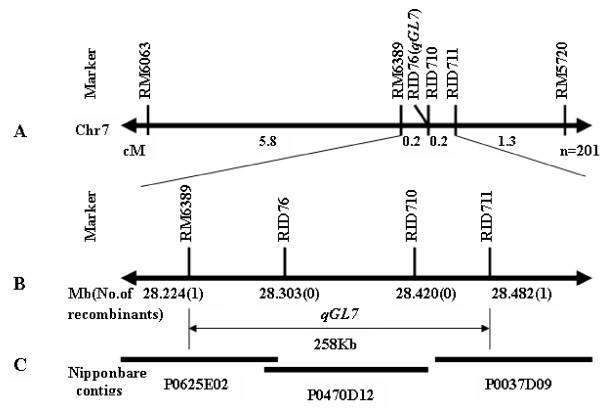
**Fine mapping of *qGL7 *and its candidate region**. (A) Local genetic map of *qGL7 *constructed with *qGL7*-NILs. (B) The physical positions of markers close linked to *qGL7*. The physical distance was determined from the sequence of Nipponbare and the known positions of the markers on the sequence. Number in parentheses indicates the number of recombinants between the marker and *qGL7*. (C) A contig consisted of the PAC, P0625E02, P0470D12 and P0037D09, contained *qGL7*.

A total of 201 individuals of the NIL-F_2 _population were genotyped by three SSR markers and three InDel markers. A local genetic linkage map of *qGL7 *region was constructed (Figure 4A). GL showed a bimodal distribution of 7.1 mm as the boundary in the F_2 _population. In contrast, GW and GT exhibited normal distributions. Significant correlations were observed between the five traits in the F_2 _population (data not shown). A positive correlation (r = 0.43, P = 0.01) between GL and GW was also detected, which is opposite to the case in the RIL population. Again, the *qGL7 *with a LOD score of 42.2 was mapped to the interval between markers RID76 and RID711, whose 1-LOD confidence interval spanned a 0.4-cM region from RM6389 to RID711. The Nanyangzhan allele increased GL and could explain 61.5% of the phenotypic variance in GL (Table 4).

**Table 4:** *qGL7 *effects on grain shape and grain yield traits in the NIL-F_2 _population

Traits	Intervals	LOD	LOD peak	Confidence interval (cM)^a^	Add^b^	Dom^c^	Var (%)^d^
GL (mm)	RID76-RID711	42.2	RID76	5.8-6.2	0.24	0.14	61.5
GW (mm)	RID76-RID711	10.6	RID76	5.0-6.3	0.04	0.04	21.5
GT (mm)	RID76-RID711	32.1	RID76	5.8-6.3	0.05	0.03	51.7
SPP(n)	RID76-RID711	15.5	RID76	4.0-6.2	-16.6	-13.4	29.3
TGW(g)	RID76-RID711	66.8	RID76	5.8-6.2	1.71	1.14	77.9

^a^Genetic position in the local linkage map (Figure 4A).

^b^Add additive effect, positive additive effect means Nanyangzhan allele increasing trait values.

^c^Dom dominance effect.

^d^Var variation explained by QTL

On the basis of progeny tests, all 201 families could be divided into three subclasses: 62 families showed identical long grain within family, 55 families showed identical short grain within family, and 84 families showed varied GL. The ratio of the number of plants in short grain and long grain groups agreed with the expected segregation of a single mendelian factor (χ^2 ^= 5.91, P > 0.05), which is indicative of one gene controlling the GL variation. These three subclasses corresponded to the three genotypes at *qGL7*, Nanyangzhan homozygotes (NN), Chuan7 homozygotes (CC), and heterozygotes (NC), respectively (Table 5). The averaged GL of NN and NC classes were 7.4 and 7.3 mm, respectively, significantly longer than that of CC class with an average GL of 6.9 mm (Figure 1). Thus, CC genotypes were easily distinguished from NN and NC. Genotypes NN and NC had similar ranges, means, and standard deviations in GL, but they were still distinguishable because the progenies from a single NC F_2 _plant segregated in GL, which was clearly evident since individual F_3 _families of this class had higher standard deviations around the mean GL than individual families of the NN class, which did not segregate. Then *qGL7*, regarded as a co-dominant marker, was mapped to the same interval of RM6389-RID711 and co-segregated with RID76 and RID710. The interval of RM6389-RID711 covered a 258-kb region in Nipponbare (Figure 4B). The Nanyangzhan allele of *qGL7 *controlling long grains was dominant against Chuan7 allele.

**Table 5:** Trait performance of the three genotypes at *qGL7 *on the basis of progeny tests

	NN (62 families)		NC (84 families)		CC (55 families)	
	
Traits	Mean ± SD	Range	Mean ± SD	Range	Mean ± SD	Range
GL (mm)	7.4 ± 0.12	7.1-7.7	7.3 ± 0.11	7.1-7.6	6.8 ± 0.15	6.5-7.0
GW (mm)	3.0 ± 0.07	2.8-3.2	3.0 ± 0.07	2.7-3.1	2.9 ± 0.07	2.8-3.1
GT (mm)	2.0 ± 0.03	1.8-2.0	2.0 ± 0.04	1.9-2.0	1.9 ± 0.04	1.8-2.0
SPP (n)	117 ± 18	90-138	115 ± 17	91-140	152 ± 22	116-201
TGW(g)	19.1 ± 0.74	17.0-21.6	18.5 ± 0.79	16.9-20.1	15.6 ± 0.82	14.3-16.4

NN homozygous for Nanyangzhan allele, NC heterozygous, CC homozygous for Chuan7 allele, GL grain length, GW grain width, GT grain thickness, SPP spikeletes per panicle, TGW 1000-grain weight

### Pleiotropic effects of *qGL7*

QTLs for the other four traits were also detected in the same interval in the NIL-F_2 _population. Their corresponding LOD peaks were exactly mapped to the maker locus of RID76/RID710 (Table 4). Their 1-LOD confidence intervals largely overlapped. A negative correlation was detected between SPP and TGW (r = -0.42, P = 0.01). That is, the panicle with long grains always had fewer secondary branches than that of the panicle with short grains (data not shown), and vice versa. The average TGWs of Nanyangzhan and Chuan7 homozygous genotypes at *qGL7 *in the F_2 _population were 19.1 g and 15.6 g, respectively. Their corresponding SPP averages were 117 and 152. The QTLs could explain 77.9% and 29.3% of the phenotypic variance in TGW and SPP. The Nanyangzhan allele increased GL and TGW but decreased SPP (Table 4). Accordingly, the progenies could be classified into three subclasses based on SPP and TGW performance: 62 families had identical small panicles with larger TGW but fewer SPP, 55 families showed identical large panicles with smaller TGW but more SPP, and 84 families showed segregation in TGW and SPP within each family. SPP was co-segregated with GL and TGW in the NIL-F_2 _population. Moreover, these three subclasses were identical to the three genotypes at *qGL7*, NN, CC, and NC, respectively (Table 5). Therefore, QTLs for SPP and TGW were co-segregated with *qGL7*.

## Discussion

### More QTLs identified in the RIL population than in previous single populations

One important finding in this study was to identify 28 QTLs for grain shape using a population with wide variations in grain shape. Although grain shape has received increased attention since the 1990s, most mapping populations used in grain shape-targeted QTL mapping studies were derived from parents whose cultivars usually produce common sized grains (TGW from 20 to 32 g) [2,7,8,11,31-34]. Populations derived from wild rice and cultivar were also used to determine the genetic basis of grain shape. Yoon et al. identified two grain size QTL hotspots located on chromosomes 6 and 11 using an advanced backcross population that was derived from wild rice, *Oryza grandiglumis *and *japonica *cultivar Hwaseongbyeo [35]. QTL analyses for grain shape showed that it was mainly controlled by *GS3*, *GW2*, *GW5*, and *GT2 *[18,19,35]. In general, a mapping population derived from two contrasting parents in grain shape is expected to detect more QTLs. In this study, the mapping population was derived from two *indica *parents with contrasting grain shape, long slender and short round. Their genetic backgrounds were also diverse (52% of investigated markers showed polymorphism) and provided us the potential to detect more QTLs for grain shape. The 28 QTLs identified in the population is a higher total than found in any other single population and proved this potentiality. Among them, two major QTLs, *qGL3a *and *qGW5a*, were repeatedly identified [11,14,15]. Also, a minor QTL, *qGW3*, for GW on chromosome 3 was also detected by Yoon et al. [35]. Interestingly, a minor QTL, *qGW2a*, identified in this study is located in the similar region of *GW2 *that was cloned by Song et al. as a major QTL [19]. This indicated that NIL populations derived from two different parental alleles at a given QTL will result increased or decreased QTL effects because different parents carry different functional alleles. Seven novel QTLs, including *qGL3b*, *qGW5b*, *qGW9b*, *qGT3a*, *qGT3c*, *qGT10b*, and *qLWR7*, were also detected in this study. Therefore, the RIL population in this study could identify more QTLs than a previous single population. In addition, besides the major QTLs and the QTL hotspots located on the long arm of chromosome 9, a minor QTL, *qGL7*, was repeatedly identified in two different environments. Other QTLs were detected only in one year, but some of them could also be detected if the LOD threshold decreased to 2.0, an empirical threshold in rice [36]. This indicated that a minor QTL could interact with the environment and was reliable only in certain conditions.

### Pleiotropic effects of QTLs

In the NIL-F_2 _population, QTLs for TGW, SPP, GW, and GT were detected in the same target region of *qGL7*. Their 1-LOD confidence intervals largely overlapped. This suggested the possibility that *qGL7 *had pleiotropic effects on all five traits. Furthermore, progeny tests showed that *qGL7 *co-segregated with the QTLs for TGW and SPP, which indicated *qGL7 *may simultaneously control GL, TGW, and SPP. Although *qGL7 *showed pleiotropic effects in the NIL-F_2 _population, a limited resolution of mapping due to the small size of F_2 _population still cannot clearly discern two closely linked genes or one pleiotropic gene in a small region. However, QTLs with pleiotropic effects on grain shape were frequently found in rice. Fan et al. reported that the *GS3 *gene had a major effect on GL and a minor effect on GW and GT in NILs [17]. Similarly, Song et al., using transgenic plants of *GW2*, a QTL that controls GW, confirmed that it also had an opposite effect on grain number per panicle [19]. Thus, *qGL7 *was a grain shape gene. Based on our current results, we tend to believe that *qGL7 *has pleiotropic effects on TGW and SPP, though in opposite directions. Moreover, due to its counteracting effects on TGW and SPP, *qGL7 *has no effect on grain yield. This implies that *qGL7 *may have an important role in balancing the distribution between SPP and TGW. That is, more distribution to SPP results in a decrease in TGW, and vice versa.

### Fine mapping and cloning of minor QTLs

Several QTLs for grain shape (grain weight) such as *gw3.1/GS3*, *gw8.1*, *gw9.1*, *GW2*, and *gw-5 *[16,17,19,22,23,37] were fine mapped in the last 5 years. So far, all cloned grain shape QTLs had major effects. Of great interest, two major QTLs, *qGL3a *and *qGW5a *in this study, shared similar positions with cloned genes *GS3 *and *qSW5*/*GW5 *[17,20,37]. It is therefore tempting to speculate that they are exactly the cloned genes and the minor QTLs of *qGL7*, commonly detected in both years, used for fine mapping. Obviously, cloning of minor QTLs detected in a primary population is more difficult. Development of QTL-based NILs revealed that most QTLs have major effects in homogeneous genetic backgrounds [38-40]. Thus, the construction of high-quality NILs became a vital step for fine mapping and cloning minor QTLs.

Rice grains are covered by hulls. The GL and GW are fixed as long as the panicle is normally differentiated. Thus, rice GL and GW are mainly controlled by genotype and have higher heritability. However, GT is greatly affected by filling degree, which is considerably affected by environment. Hence, for pleiotropic QTLs for grain shape, GL and GW provided more reliable trait values for gene cloning. Although the minor QTL, *qGL7*, explained only about 5% of GL variation in RILs, it clearly expressed the character of a single mendelian factor and explained most parts of the trait variation in the NIL-F_2 _population. It is promising to narrow down *qGL7 *to a small region given a large NIL-F_2 _population. Characterization of *qGL7 *clearly indicated that a minor QTL can be treated as a single mendelian factor for further functional research on quantitative traits especially when major QTLs have been cloned.

### Molecular breeding design for rice grain appearance improvement

Grain appearance, which is mainly evaluated by the parameter of LWR, is an essential component of rice quality. Comprehensive understanding of the genetic basis of grain shape will be particularly helpful in improving its appearance quality. No transgressive segregation was observed for GL, GW, and GT in RILs, which indicated that positive alleles were almost all from Nanyangzhan. Actually, among the 28 QTLs for GL, GW, and GT, only at two minor QTLs of parental Chuan7 were trait values increased. On the other hand, positive alleles were not always favored in rice improvement. For example, consumers in East Asia prefer short and round rice. Especially long and slender grains (large GL and large LWR) always decreased head rice percentage when rice was milled. In our study, 4 and 10 QTLs were identified for GL and GW, respectively. The major QTLs were, of course, first considered for improving grain shape. For example, if we focused on the four QTLs of *qGL3a*, *qGL7*, *qGW5a*, and *qGW9b*, their combinations can produce 16 homozygous genotypes. If the plant fixed with Nanyangzhan alleles at *qGL3a *and *qGL7*, and Chuan7 alleles at *qGW5a *and *qGW9b*, its ratio of GL/GW would produce the largest LWR of 3.86; however, in the reciprocal case, the ratio would change to the smallest LWR of 2.26 (Table 6). Among the 16 genotypes, a diverse LWR variation, ranging from 2.26 to 3.86, provides breeders flexibility in designing new varieties for people's preference in different regions. The flanking markers of the four QTLs can be directly used for marker-aided selection.

**Table 6:** Ratios of GL to GW in the gene combinations among four grain shape QTLs

Genotypes	*qGW5aqGW9b*			
***qGL3aqGL7***	**NNNN**	**NNCC**	**CCNN**	**CCCC**

NNNN	3.36	3.43	3.64	3.86
NNCC	2.83	2.89	3.06	3.25
CCNN	2.48	2.53	2.68	2.84
CCCC	2.26	2.30	2.44	2.59

NN homozygous for Nanyangzhan allele, CC homozygous for Chuan7 allele.

## Conclusion

In this study, we performed QTL analysis for grain shape using an RIL population derived from two varieties with contrasts in grain shape. Twenty-eight QTLs were identified. Seven of them were detected for the first time. These results demonstrated that the mapping population derived from parents with contrasting phenotypes can be used for detecting more QTLs. Diverse materials are recommended to develop mapping populations for powerful QTL detection. In the present study, a minor QTL of *qGL7 *was validated with pleiotropic effects on GL, GW, TGW, SPP, and GT in an NIL-F_2 _population. It is suggested that minor QTL of a highly heritable trait could be isolated following the strategy of map-based cloning in a large NIL-F_2 _population. Combinations of different QTLs were expected to produce diverse grain shapes from long slender to short and round rice, which provides more flexibility to breed rice varieties for different preferences.

## Authors' contributions

BXF conducted the experiment, developed the genetic linkage map, and analyzed the data. LLJ developed the RIL population. YWH, KMR, and ZW partially contributed in phenotypic data collection and population genotyping. XYZ designed the work and wrote the manuscript together with BXF.

All authors have read and approved the final manuscript.
